# CXC Chemokine CXCL12 and Its Receptor CXCR4 in Tree Shrews (*Tupaia belangeri*): Structure, Expression and Function

**DOI:** 10.1371/journal.pone.0098231

**Published:** 2014-05-23

**Authors:** Guiyuan Chen, Wei Wang, Shengke Meng, Lichao Zhang, Wenxue Wang, Zongmin Jiang, Min Yu, Qinghua Cui, Meizhang Li

**Affiliations:** 1 Department of Biochemistry & Molecular Biology, School of Life Sciences, Yunnan University, Kunming, China; 2 Department of Biochemistry & Molecular Biology, School of Basic Medicine, Dali University, Dali, China; 3 Department of Rheumatology & Immunology, First Affiliated Hospital of Kunming Medical University, Kunming, China; 4 Department of Hepatobiliary Surgery, Second Affiliated Hospital of Kunming Medical University, Kunming, China; University of Leuven, Rega Institute, Belgium

## Abstract

Chemokines are small secreted proteins functionally involved in the immune system's regulation of lymphocyte migration across numerous mammalian species. Given its growing popularity in immunological models, we investigated the structure and function of chemokine CXCL12 protein in tree shrews. We found that CXCL12 and its receptor CXCR4 in tree shrew had structural similarities to their homologous human proteins. Phylogenetic analysis supports the view that tree shrew is evolutionarily-close to the primates. Our results also showed that the human recombinant CXCL12 protein directly enhanced the migration of tree shrew's lymphocytes *in vitro*, while AMD3100 enhanced the mobilization of hematopoietic progenitor cells (HPCs) from bone marrow into peripheral blood in tree shrew *in vivo*. Collectively, these findings suggested that chemokines in tree shrews may play the same or similar roles as those in humans, and that the tree shrew is a viable animal model for studying human immunological diseases.

## Introduction

Animal models can effectively demonstrate some of the complexities of both human diseases and the human immune system [1]. While mice and rats have been traditionally used as effective low-cost models, the use of the tree shrew has recently gained in popularity. Tree shrews (*Tupaia belangeri*) are small animals, mainly distributed in South Asia, Southeast Asia and Southern China [2]. Unlike primates, tree shrews are relatively cheap and simple to handle, and although phylogenetic analysis of mitochondrial DNA sequences has shown a close relationship between tree shrews and lagomorphs/rodents [3]–[5], recent studies using whole genomic sequences have suggested that tree shrews are more closely related to primates than to rodents [6].

While different studies have provided conflicting reports on the origin of the tree shrew as well as its relationship to primates[6], [7], tree shrews have still consistently been used to develop experimental models for studying human viruses such as hepatitis A, B, C and H1N1 [8]–[12]. The success of these efforts has largely stemmed from the conceivable similarity between the immune system of the tree shrew and humans (which is not fully characterized, yet) as well as the structural evolution of genes at work in the immune system shared between tree shrews and other primates [6], [13]. Accordingly, the next step in assessing the viability of the tree shrew model for immunological studies is to gain a more comprehensive understanding of the tree shrews' molecular and cellular immune mechanisms.

Within the vertebrate immune system, chemokines are small secreted proteins responsible for regulating leukocyte trafficking during host defense immune response [14]. Chemokines are generally classified into four subfamilies–CXC, CC, C and CX3C–according to two conserved cysteine (C) residues at their N-terminal protein sequences [15], [16]. The chemokine CXCL12 protein (also known as stromal-derived factor 1, SDF-1) is an important member of the CXC chemokines [16] with two spliced variants: CXCL12 has two spliced variants CXCLl2α (89 amino acids) and CXCLl2β (93 amino acids) [17]. Two receptors, CXCR4 and CXCR7, have previously been found to be bound by CXCL12 with high specificity and affinity [18]. Both receptors are typical G-protein-coupled receptors (GPCRs) with seven-transmembrane domains [19]–[22], but only CXCR4 can activate G-protein-mediated downstream signaling pathways [23], [24]. The CXCR4-specific antagonist AMD3100 inhibits the signal transduction induced by the ligand CXCL12 [25].

The CXCL12-CXCR4 axis is well established to play multiple roles during cellular migration, survival and proliferation, as well as in other functions. [26]–[29]. CXCL12/CXCR4 signaling also has been implicated as important axis in the bone marrow niche, regulating not only retention but also migration and mobilization of HPCs [30]. Given these functions, the CXCL12-CXCR4 axis represents a structurally- and functionally-conserved signaling pathway in the immune system [16]. Unfortunately, their basic structure and function in tree shrews remains unclear, which greatly complicates efforts to utilize this species as an effective immunological model. To explore both the structure and function of this axis, we cloned the tree shrews CXCL12 and used real-time polymerase chain reaction (RT-PCR) and bioinformatics to characterize both it and its receptor CXCR4. We found that CXCL12 and CXCR4 had structural similarity to their homologous human proteins and that in tree shrews CXCL12-CXCR4 chemotaxis is necessary to regulate the migration of peripheral lymphocytes similar to the role played in humans [29]. Furthermore, AMD3100 enhanced the mobilization of HPCs from bone marrow into peripheral blood in tree shrew *in vivo,* suggesting that the CXCL12–CXCR4 signaling plays a pivotal role in the egress of HPCs from bone marrow into peripheral blood in tree shrews. These findings suggest that the CXCL12-CXCR4 axis observed in tree shrews is a highly-conserved system across mammals and may play important roles during physical and pathological immune responses. These results provide novel evidence that supports the growing use of tree shrews as a model for human immunological studies and diseases.

## Results

### Cloning of CXCL12 full length coding sequence (CDS)

**Figure 1:**
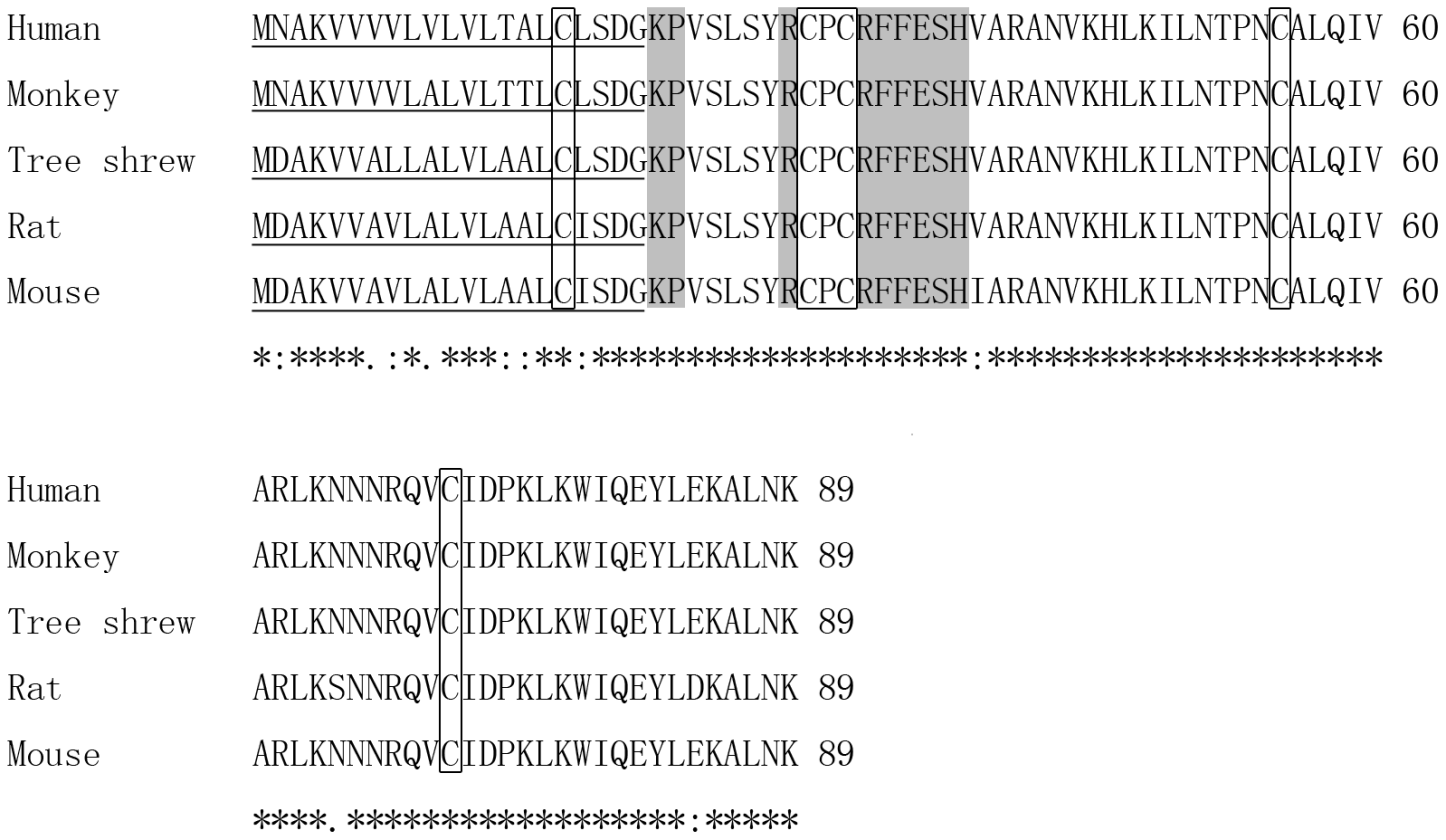
Alignment of five different CXCL12 proteins. Signal peptide sequences are underlined. CXC sites and conserved cysteines are boxed. CXCR4-binding domains are shadowed. Identical amino acid residues are marked by an asterisk. Conserved and semi-conserved residues are indicated by a colon and period respectively.

The coding sequence of CXCL12 was initially cloned using tree shrew brain tissue samples. Following cloning, analysis showed that the sequence contained a 270 nucleotides (nt) sequence (accession number KF640640), predicting an small 89-amino-acid (aa)-length small protein (Fig. S1). This predicted protein included a totally-conserved structure organization. Similar to that in other mammalian species, the tree shrew CXCL12 protein includes two parts: a 21 aa signal peptide part “MDAKVVALLALVLAALCLSDG” and a 68 aa main part (Fig. 1). Similar to its human homologue, a typical “KPVSLSYRCPCRFFESH” sequence was also found at the N-terminal site of the tree shrew CXCL12 protein, and it is this conserved 17 aa domain that directly interacts with the receptor CXCR4 in humans [31].

### CXCL12 and CXCR4 proteins in tree shrews are highly homologous to those in humans

**Figure 2:**
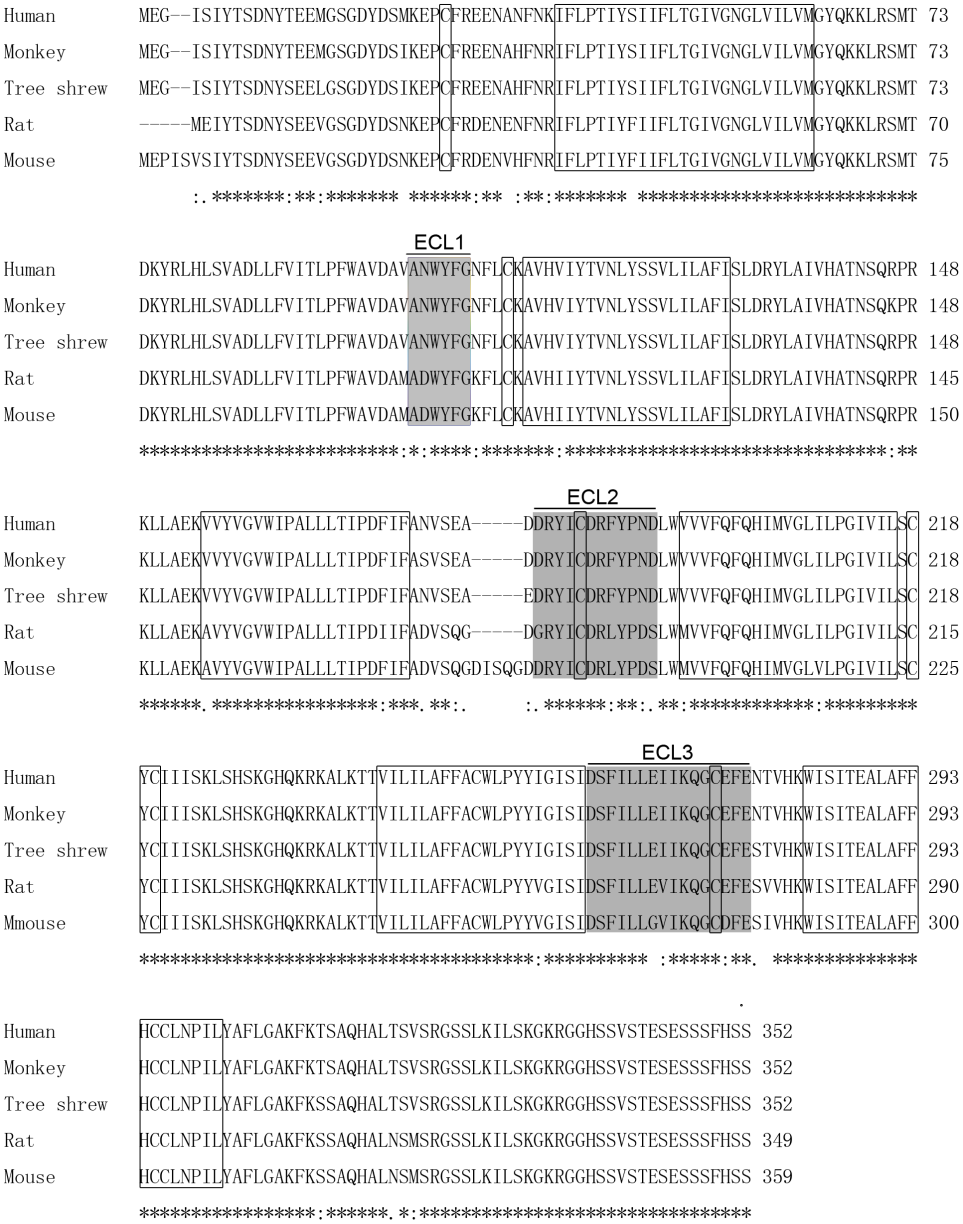
Alignment of five different CXCR4 proteins. Seven-transmembrane domains and conserved cysteines are boxed. Extracellular loops are shadowed and marked with the first, second and third extracellular loops (ECL1, ECL2 and ECL3, respectively). Identical amino acid residues are marked by an asterisk. Conserved and semi-conserved residues are indicated by a colon and period respectively. Dashes indicate gaps introduced into the sequences to optimize alignment.

To explore the evolutionary trajectory of CXCL12 proteins in tree shrews, we compared the CXCL12 protein sequences between five different mammals: humans, monkeys, tree shrews, rats and mice. Our comparative analysis found a highly homologous amino acid sequence of CXCL12 between tree shrews and the other tested mammals. In particular, the CXCL12 protein of the tree shrew exhibited 94.4% sequence homology to human and monkey CXCL12 amino acid sequences, and 97.5% and 95.5% homology to mouse and rat CXCL12 amino acid sequences, respectively (Fig. 1). We further analyzed the CXCR4 protein of the tree shrew according to a 1059 nt sequence previously deposited in GenBank (accession number: AY177628.2). The CDS predicted a 352 aa sequence (Fig. S2). Alignment of CXCR4 between the five mammal species also showed a highly-conserved amino acid sequence (Fig. 2). The tree shrew CXCR4 protein contained seven-transmembrane domains and three extracellular loops 1-3 (ECL1-3) (Fig. 2). Further analysis showed the similarities between the CXCR4 proteins of tree shrews and those of humans and monkeys were as high as 97%, and those of mice and rats were 91% and 92%, respectively.

**Figure 3:**
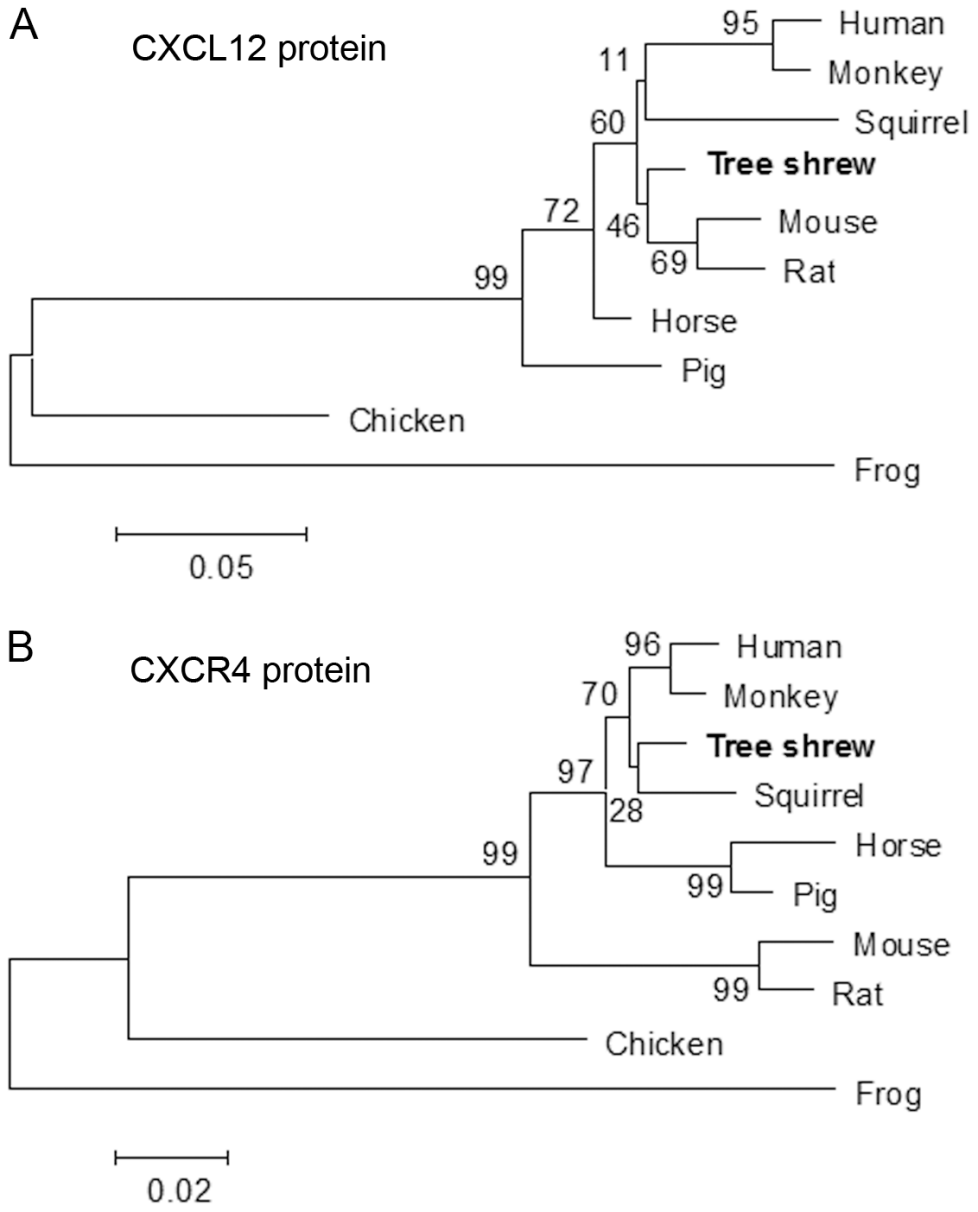
Phylogenetic analysis of ten different CXCL12 and CXCR4 proteins. (**A**) Unrooted phylogenetic tree of CXCL12 proteins. (**B**) Unrooted phylogenetic tree of CXCR4 proteins. Phylogenetic trees were constructed using full length amino acid sequences with Neighbor-joining method and Maximum likelihood method within MEGA 5.0 and bootstrapped 1000 times.

Phylogenetic analysis of the CXCL12 proteins from ten different species demonstrated that tree shrews were first clustered with mice and rats, and secondly with humans and monkeys (Fig. 3A). Interestingly, varied amino acids were only observed in the region of the signal peptide. The main secreted part of the tree shrew CXCL12 protein was identical to that of the human CXCL12 protein (Fig. 1). The CXCR4 phylogenetic tree for the ten compared species also consistently grouped tree shrews and squirrels directly with humans and monkeys (Fig. 3B). These results suggest that the CXCL12 and CXCR4 proteins in tree shrews have a conserved structure and amino acid sequence.

### Three-dimensional structures of CXCL12 and CXCR4 in tree shrews

**Figure 4:**
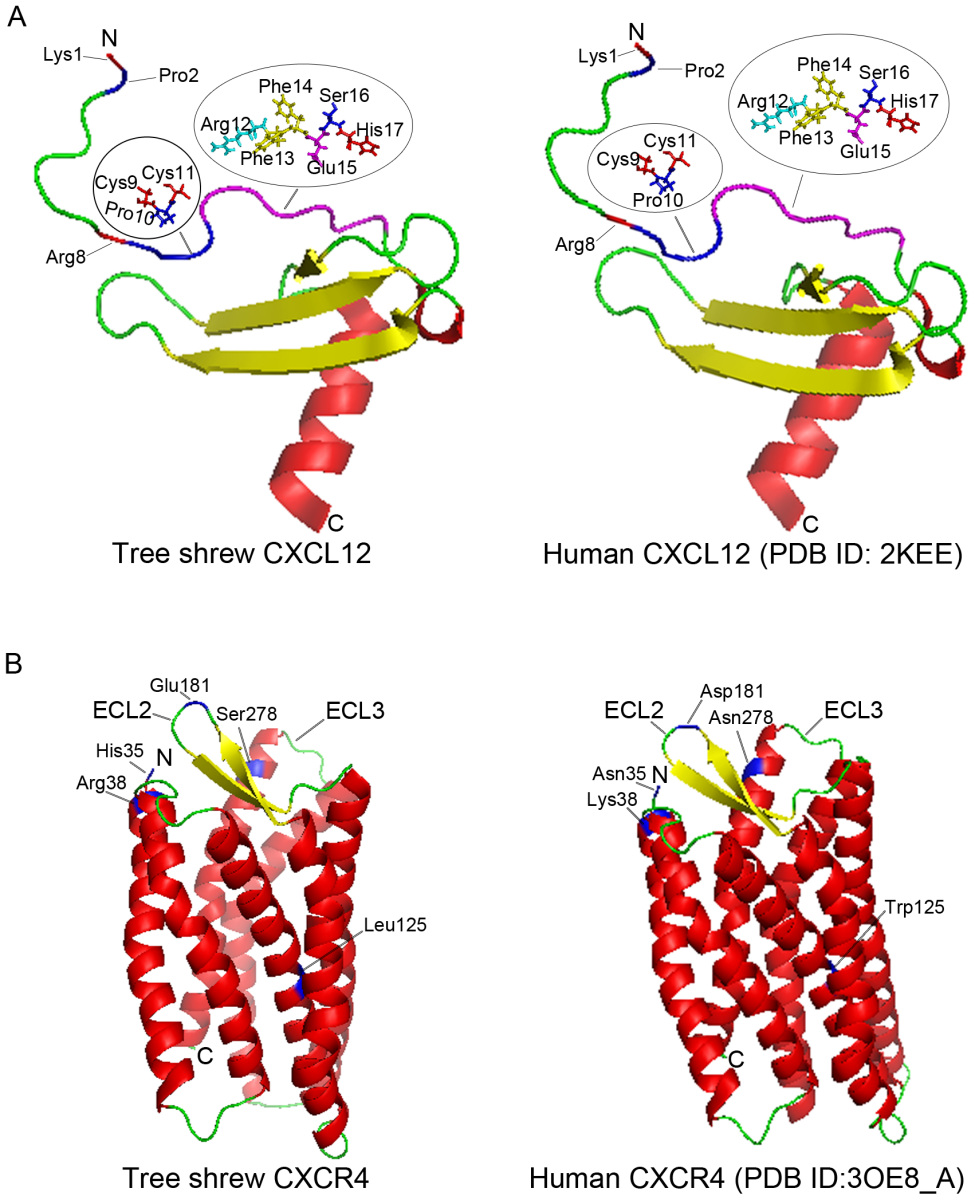
Three-dimensional structures of CXCL12 and CXCR4 proteins. (**A**) 3D Structure of tree shrew CXCL12 (left) compared with human CXCL12 (right). CXCR4-binding domains are in purple, including sequence “Arg12-Phe13-Phe14-Ser15-Glu16-His17”. Three individual functional amino acid residues, which bind to CXCR4 and regulate the molecular conformation of CXCR4, are marked by Lys1 (red), Pro2 (blue) and Arg8 (red). Conserved Cys9-Pro10-Cys11 site is in blue. (**B**) 3D structure of tree shrew CXCR4 (left) compared with human CXCR4 (right). Conserved seven-transmembrane α-helix domains are in red. Second extracellular loop (ECL2) and third extracellular loop (ECL3) are in green. Semi-conserved amino residues on the structure of CXCR4 between tree shrew and human are in blue. N-terminal (N), C-terminal (C).

The finding of a conserved amino acid sequence in the CXCL12 and CXCR4 proteins of tree shrews prompted further prediction of their three-dimensional (3D) protein structures via Homology Modeling (HM), using human CXCL12 and CXCR4 crystal structures as the templates. Similar to the CXCL12 protein of humans, the structure of the tree shrew CXCL12 protein was composed of two α-helixes, three anti-parallel β-sheets and four loops. The CXCR4- binding sequence KPVSLSYR-CPC-RFFESH, which exists in the CXCL12 protein of humans, was also found in tree shrew CXCL12 protein (Fig. 4A) [31]. Similar observations of the 3D structures showed that the CXCR4 proteins of both tree shrews and humans consisted of seven-transmembrane α-helixes, two anti-parallel β-sheets and three extracellular loops (Fig. 4B). The functional amino acid residues of CXCR4 located in ECL2 (Asp182, Tyr184, Asp187, Arg188, Tyr190 and Asp193) and ECL3 (Asp262, Glu268 and Glu277) are bound by CXCL12 in humans [32]–[35]. We found identical amino acid residues in ECL2 and ECL3 of the CXCR4 protein in humans and tree shrews. The semi-conserved amino acid residues in the structure of the CXCR4 protein in humans and tree shrews did not locate in functional domains. These findings indicated a similar binding relationship between the CXCL12 and CXCR4 proteins in tree shrews as found in humans.

### Expression of CXCL12 and CXCR4 in tissues of tree shrews

**Figure 5:**
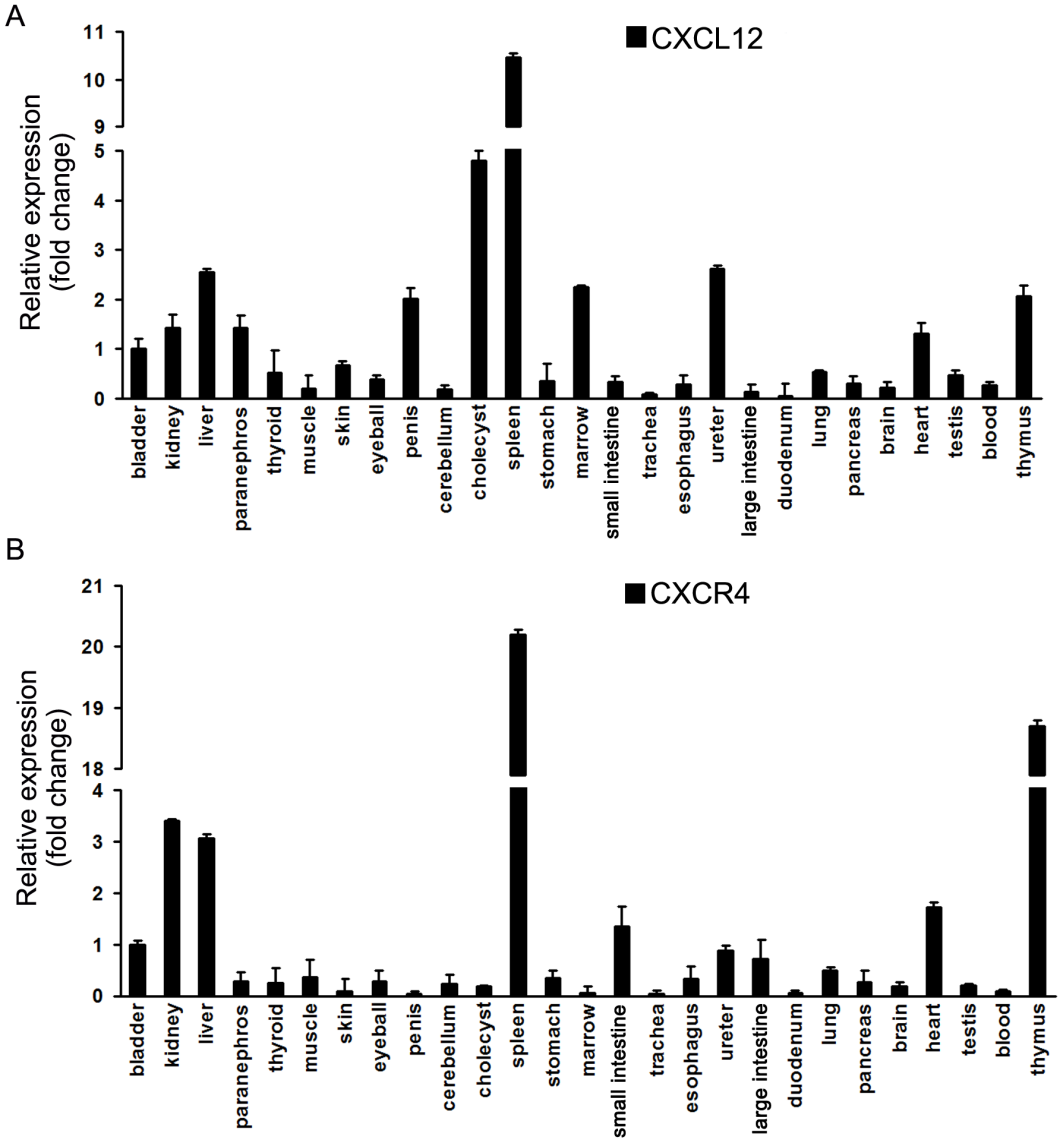
Tissue-specific mRNA expression of tree shrew's CXCL12 and CXCR4. Expression of CXCL12 (**A**) and CXCR4 (**B**). The expression levels of CXCL12 and CXCR4 were detected using Real time-PCR.

We examined the expression levels of CXCL12 and CXCR4 mRNAs in 27 different tree shrew tissues (bladder, kidney, liver, paranephros, thyroid, muscle, skin, eyeball, penis, cerebellum, cholecyst, spleen, stomach, marrow, small intestine, trachea, esophagus, ureter, large intestine, duodenum, lung, pancreas, brain, heart, testis, blood and thymus). Our results illustrated a varied and tissue-dependent gene expression spectrum for the CXCL12 and CXCR4 genes (Fig. 5A and 5B), with high expression levels in the tissues of kidney, liver, spleen, heart and thymus. In some tissues (e.g., thyroid, muscle, skin, eyeball, stomach, trachea, esophagus, duodenum, lung, pancreas, testis, etc.) both genes showed relatively-low expression levels. CXCL12 exhibited a relatively-high expression in the tissues of paranephros, penis, cholecyst, marrow and ureter, but low expression in the small intestine. Intriguingly, compared with CXCL12 expression, CXCR4 was inversely expressed in these tissues (paranephros, penis, cholecyst, marrow, ureter and small intestine). We also found that CXCL12 and CXCR4 were both relatively-high expressed in immune tissues, such as spleen and thymus (Fig. 5A and 5B), implying that CXCL12 and CXCR4 may be involved in some aspect of immunological regulation among tree shrews.

### CXCL12 protein level in tree shrews blood

**Figure 6:**
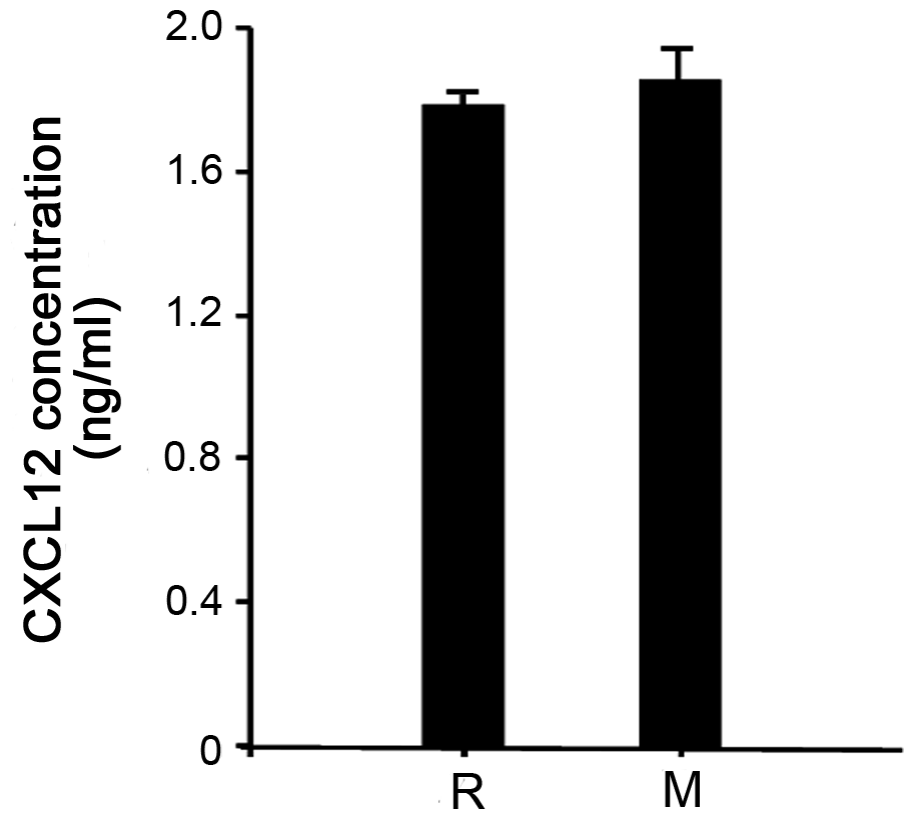
Detection of CXCL12 concentration in tree shrew serum. ELISA was conducted using mouse anti-rat CXCL12 antibody (R) and mouse anti-mouse CXCL12 antibody (M).

To determine the protein level of CXCL12 in normal tree shrew blood, we conducted a crossing-specie enzyme-linked immunosorbent assay (ELISA) using two mouse monoclonal antibodies, anti-rat (R) and anti-mouse (M) [36]. These two antibodies detected a similar concentration of CXCL12 in the serum of tree shrews. The serum CXCL12 level detected by either anti-rat (R) and anti-mouse (M) antibodies was 1.78 ng/ml and 1.85 ng/ml respectively (Figure. 6).

### CXCR4 expression in the lymphocytes in tree shrews

**Figure 7:**
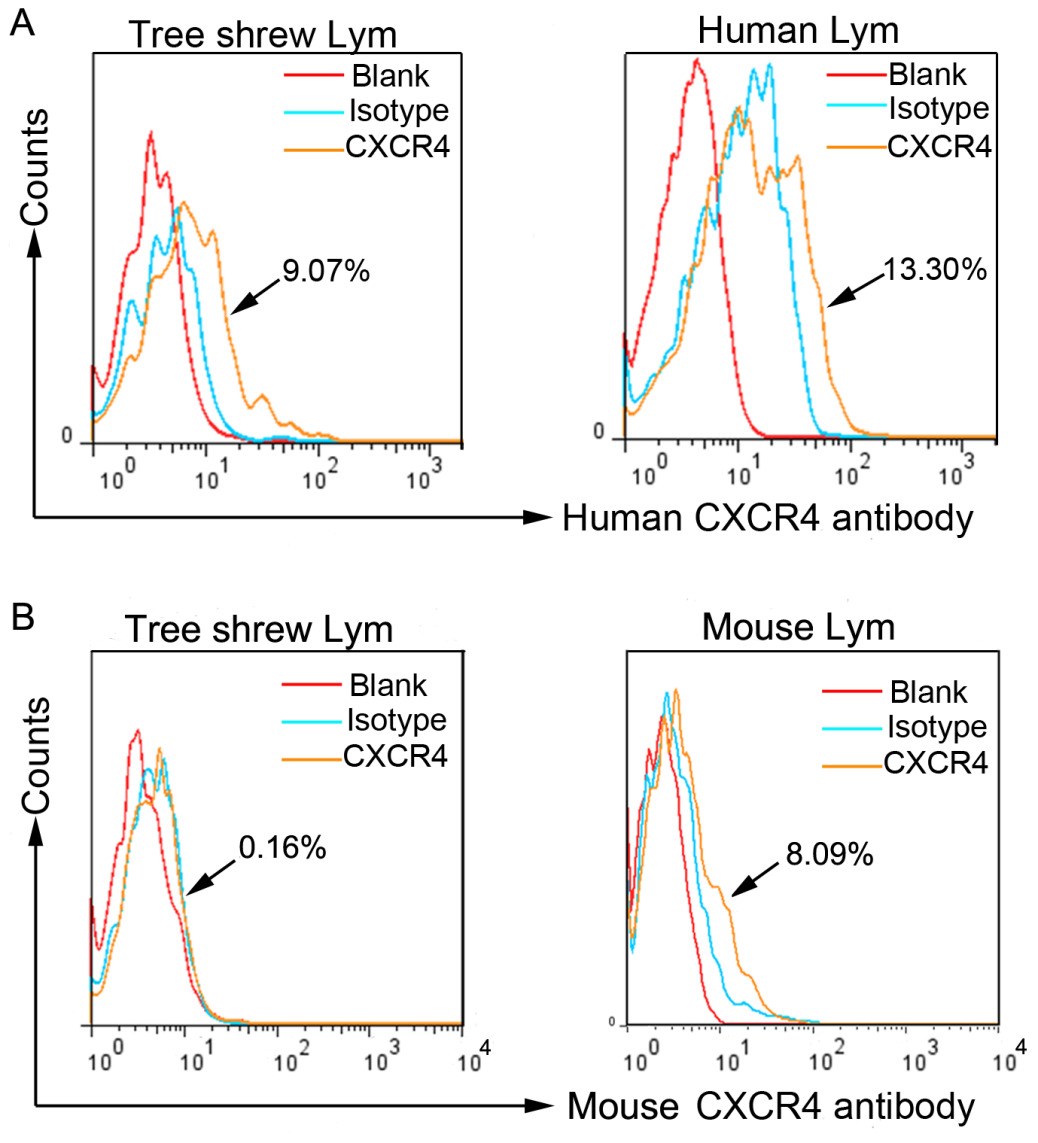
Expression of CXCR4 in tree shrews' lymphocytes. (**A**) 9.07 % of tree shrew CXCR4-positive lymphocytes were detected by mouse anti-human CXCR4 antibody (left). For the control, 13.30 % of human CXCR4-expressing lymphocytes were detected by the same antibody (right). (**B**) 0.16 % of tree shrew CXCR4-positive lymphocytes were detected by rat anti-mouse CXCR4 antibody (left). For the control, 8.09 % of mouse CXCR4-expressing lymphocytes were detected using the same antibody (right). Blank (red), isotype (green), anti-human CXCR4 antibody (brown) and anti-mouse CXCR4 antibody (brown).

We examined the protein expression level of CXCR4 in the peripheral blood lymphocytes. In a sample of tree shrew blood, we performed a crossing-species flow cytometry using two mouse monoclonal antibodies, anti-human (H) and anti-mouse (M) [37], [38]. The mouse anti-human (H) CXCR4 antibody recognized the same peptide of CXCR4 in tree shrews. This antibody detected that 9.07 % of lymphocytes expressed CXCR4 (Fig. 7A left). As a control, the human CXCR4 antibody (Fig. 7A right) detected that 13.3% of lymphocytes were CXCR4-positive. While the mouse anti-mouse CXCR4 antibody detected no peptide of CXCR4 in tree shrews, 8.09 % of lymphocytes in mouse peripheral blood expressed CXCR4, as detected by the mouse anti-mouse CXCR4 antibody (Fig. 7B right), while 0.16 % of lymphocytes were detected to express CXCR4 in the peripheral blood of tree shrews using the same antibody (Fig. 7B left).

### *In vitro* transmigration of lymphocytes by CXCL12 chemotaxis

**Figure 8:**
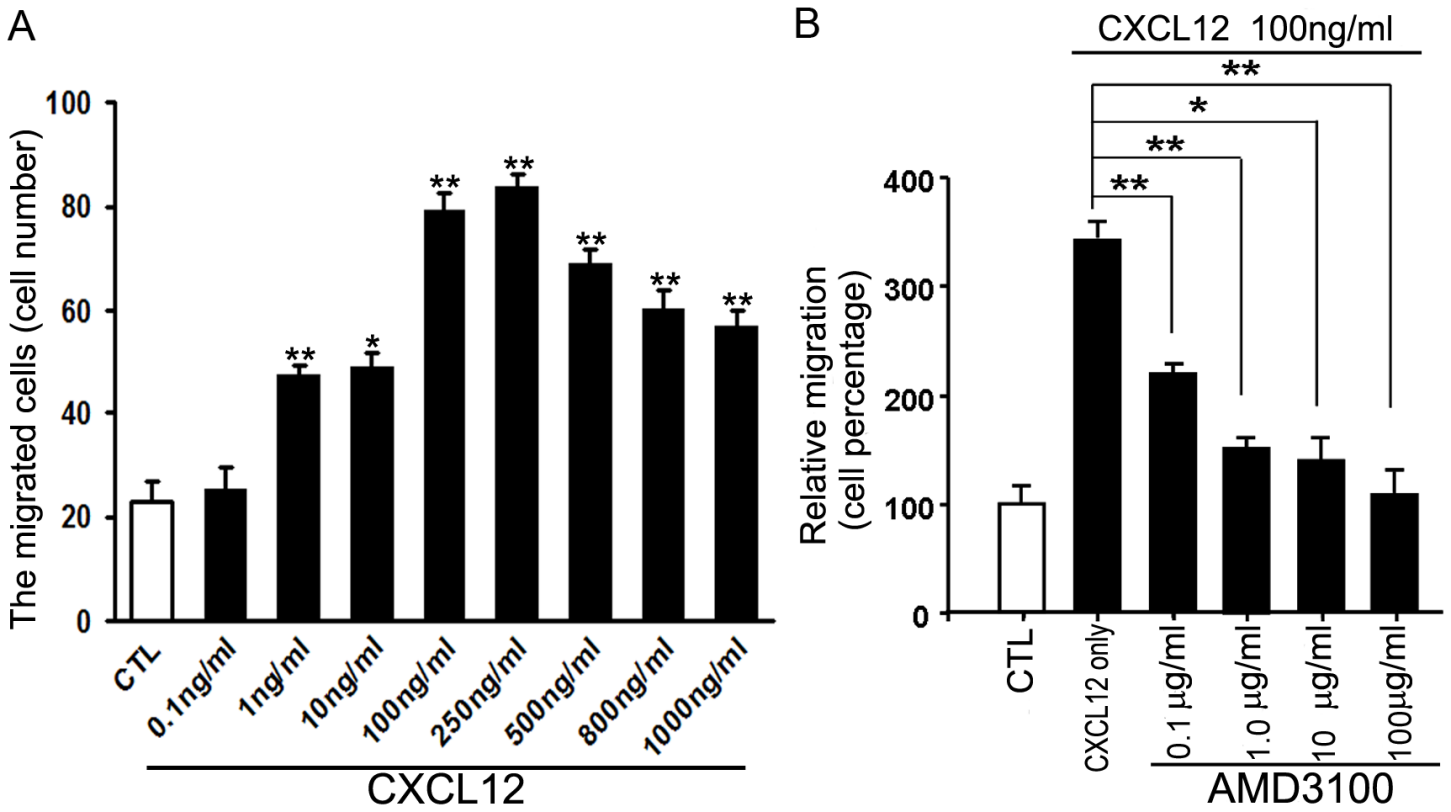
CXCL12 required for the transmigration of tree shrew lymphocytes. (**A**) Human recombinant CXCL12 induced the transmigration of tree shrew lymphocytes *in vitro*. (**B**) CXCR4-specific antagonist AMD3100 inhibited CXC12-induced transmigration of tree shrew lymphocytes. CTL (normal control), **P<0.01, *P<0.05 (n = 3).

Expression of CXCL12 and CXCR4 in lymphoid organs indicated that CXCL12-CXCR4 chemotaxis may have conserved functions manifested in the tree shrew′s immune system,such as the chemotatic roles of the CXCL12-CXCR4 signaling pathway for lymphocytes in tree shrews. We performed a transmigration assay of lymphocytes using a human recombinant CXCL12 protein as the exogenous chemokine and found that human recombinant CXCL12 affected the transmigration of tree shrew lymphocytes (Fig. 8A) in response to serial concentrations of CXCL12, with the highest migration of lymphocytes at 250 ng/ml while CXCL12 concentrations of 500 to 1000 ng/ml inhibited migration.

We further determined whether CXCR4-specific antagonist AMD3100 inhibited CXCL12-induced transmigration of lymphocytes. Results showed that 0.1 µg/ml∼100 µg/ml of AMD3100 efficiently blocked the transmigration of lymphocytes in tree shrews induced by 100 ng/ml of human recombinant CXCL12 (Fig. 8B). These findings indicated that chemokine CXCL12 signaling was functionally required to regulate the migration of peripheral blood lymphocytes in tree shrews.

### AMD3100 did not significantly decrease the survival of tree shrews' lymphocytes

To exclude potential cell toxicity induced by AMD3100, we tested the effects of AMD3100 on the survival of lymphocytes via MTT assay. The result showed that compared with the control, the survival of lymphocytes were not significantly affected by the concentration range of 0.1∼100 µg/ml of AMD3100 (Fig. S3).

### Mobilization of HPCs in tree shrews by AMD3100

**Figure 9:**
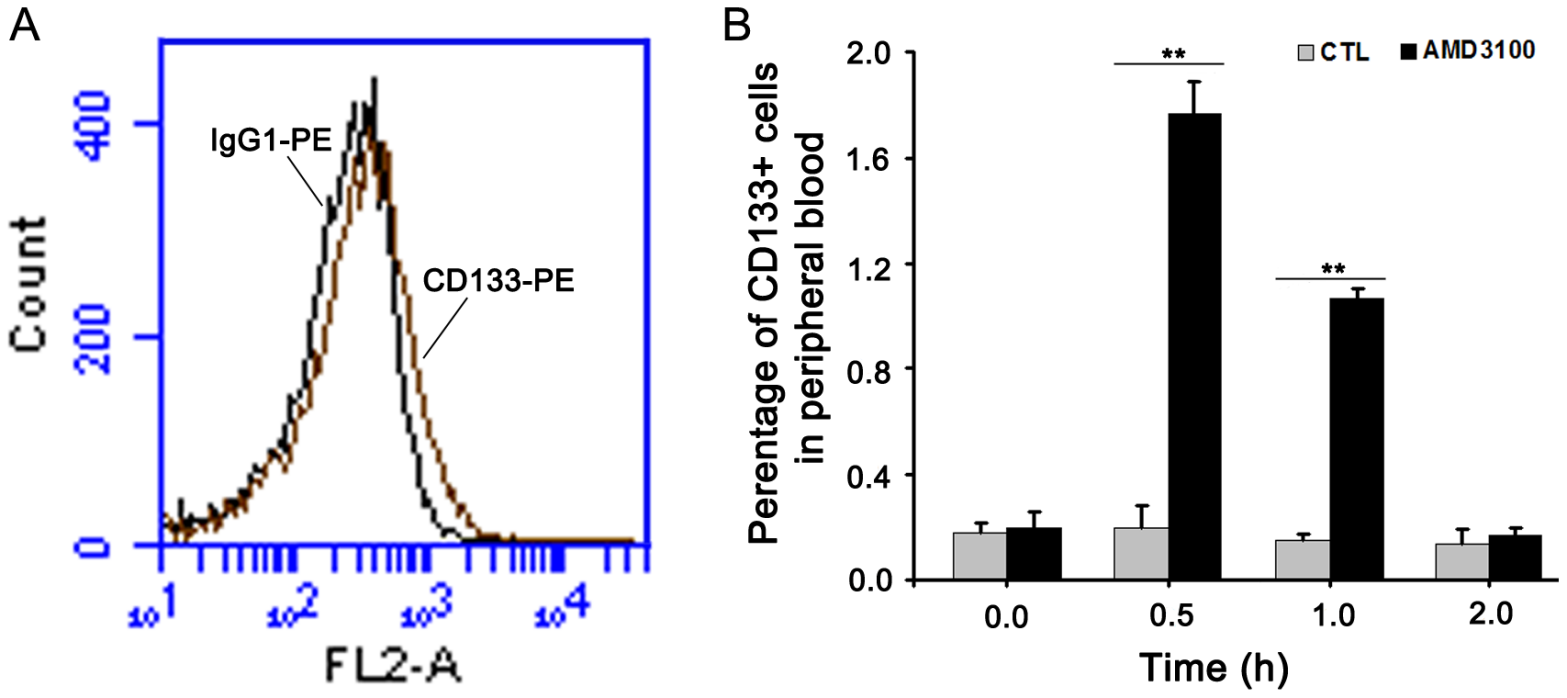
AMD3100 enhanced the mobilization of CD133+ cells *in vivo*. (A) CD133+ cells in normal peripheral blood of tree shrews were analyzed by FACS. (B) In ADM3100-treated tree shrews and PBS-treated tree shrews (CTL), peripheral CD133+ cells were analyzed at four different time points, including 0, 0.5, 1.0 and 2.0 hours respectively. **P<0.01(n = 3).

Previous studies showed that CXCL12/CXCR4 signaling is an important axis in the bone marrow niche, not only regulating retention but also migration and mobilization of HPCs [30], [39]. To test whether the CXCL12-CXCR4 axis can functionally maintain and mobilize HPCs in tree shrew bone marrow, the CXCR4 antagonists, AMD3100 was injected into tree shrews to examine the mobilization of HPCs, with FACS being used to analyze the percentage of CD133+ HPCs in the peripheral blood of tree shrews. Results showed that the percentage of CD133+ cells in the peripheral blood was up to 1.8% and got the highest mobilization of HPCs at 0.5h after administration. Thereafter, the percentage of CD133+ cells had persistently declined, and by 2.0 h after injection, the percentage of CD133+ cells fell to the normal baseline of physiological levels again (Fig. 9). These in vivo data strongly indicate that AMD3100 has the ability to mobilize HPCs from the bone marrow to the peripheral blood in tree shrew. If so, this result would strengthen the case for the importance of the participation of the CXCL12-CXCR4 axis in regulating the retention and mobilization of HPCs in the bone marrow of the tree shrews.

## Discussion

CXCL12-CXCR4 axis is a structurally- and functionally-conserved signal pathway that exists in a variety of mammalian species [40]. To date, a number of studies have reported multiple functions related to CXCL12-CXCR4 chemotaxis. CXCL12 appears to be directly involved in the inflammatory reaction during the pathological conditions [41], [42]. In mammalian bone marrow, niche-dependent stromal cells secrete the CXCL12, regulating the homing of hematopoietic stem cells [43]. CXCL12 is also highly expressed in the bone marrow around the endosteum and acts as a retention factor to maintain HPCs in the bone marrow, while the expression of CXCR4 in HPCs is essential for stem cell quiescence [44]. An antagonist of CXCR4, AMD3100, has likewise been shown to block CXCL12/CXCR4 interaction and in doing so enhance mobilization of progenitor cells from bone marrow to peripheral blood [45]. The chemokine receptor CXCR4 is likewise an important co-receptor in HIV infection [4], [46]. Recent studies have also found that CXCL12-CXCR4 chemotaxis regulates the invasion and metastasis of malignant solid tumors [22], [42], [47].

In this study, for the first time we conducted a thorough survey of the structure and function of CXCL12 and CXCR4 in tree shrews. We cloned and identified a 270 nt-length CXCL12 CDS fragment that expressed a potential 89 aa secreted protein. Bioinformatics showed that CXCL12 and CXCR4 protein in tree shrews had conserved organization and structure, similar to their homologous proteins in humans. We also found that the main secreted part of the tree shrew CXCL12 protein was identical to the CXCL12 protein in humans. According to the amino sequence alignment data of CXCL12 and CXCR4 between tree shrews and other species, phylogenetic analysis strongly supported an evolutionarily close relationship between tree shrews and primates. Even the spatial interaction of CXCL12 and CXCR4 are highly-conserved between tree shrews and humans, supporting the notion that tree shrews may actually make a viable and potentially more informative animal model than many of those currently in use.

The CXCL12 -CXCR4 axis is a structurally- and functionally-conserved signal pathway that has been researched clearly in human [40], [48]. Previous studies found that several signal transduction pathways were activated by the CXCL12-CXCR4 axis, including the PI3K/AKT, MEK/MAPK p42/44, and JAK/STAT axes, and the activation of these pathways has been shown to regulate locomotion, chemotaxis, adhesion and secretion of CXCR4 positive cells [48].Our results indicated that CXCL12-CXCR4 signal pathway in tree shrews may share similar structures and functions with their human counterparts—a necessary factor in ascertaining the viability of the tree shrew for further animal model studies. However, to date the CXCL12-CXCR4 signal pathway in the tree shrew remains unclear, and is an area of interest for future study.

Chemokines indeed play many important roles in the immune system [49]. To better understand the potential roles of CXCL12 in the immune system of tree shrews—and by extension, to help clarify how results from tree shrew models are applicable to human immunological responses—we studied the protein expression and chemotatic role of CXCL12 in peripheral blood. Our results showd that 1.8 ng/ml of the CXCL12 protein was detected in tree shrew blood. 9.07 % of tree shrew lymphocytes expressed CXCR4 on the cellular surface, and CXCL12 was functionally required to regulate the migration of CXCR4-expressing peripheral blood lymphocytes in tree shrews. Moreover, tests showed that AMD3100 was able to enhance the mobilization of HPCs from bone marrow into peripheral blood in tree shrews, suggesting that CXCL12-CXCR4 signaling participates in some form of regulating retention and mobilization of HPCs in bone marrow. Together these findings suggest that CXCL12 was not only conserved in its structure, but more importantly, that it plays several potential roles in the tree shrew immune system. Given the increase in studies on the development of tree shrews as experimental animals for virus infection, these are critical findings that clarify much that is unknown regarding the tree shrew immune system.

Current studies have provided preliminary evidence to support tree shrews as important animal models for investigating immunological diseases [8]–[12]. However, before such models can be widely accepted and applied, the detailed roles of chemokines during pathological processes must be further clarified. For example, we are currently constructing a model of rheumatoid arthritis (RA) of tree shrews to research the function of CXCL12-CXCR4 axis and determine what role of this axis may plays in the mechanisms underpinning RA. This model is just one of many being done by immunologists and other researchers working with tree shrews, all of which would benefit from clearer results on the relationship between tree shrew and human immunological mechanisms and responses, like those of the CXCL12-CXCR4 axis. To that end, hope this current study will be of use in developing further detailed investigations that will continue to support the tree shrew's usage as a valuable immunological model.

## Materials and Methods

### Ethics statement

All tree shrews used in this study were purchased from the Kunming Institute of Zoology and Kunming Medical University. All animal experimental protocols were viewed and approved by the medical ethics committee of the School of Medicine, Yunnan University, Yunnan province, China.

### Animal breeding and isolation of tree shrews tissues

During the experiment, tree shrews were freely fed with tap water and mixed provender and were housed in individual cages. Once they reached four months of age, five healthy tree shrews were anaesthetized by ether in a hermetic case for five minutes in order to collect peripheral blood via cardiac puncture. These tree shrews were then euthanized by carbon dioxide for dissection [50], [51]. The varying studied tree shrew tissues were removed under sterile conditions, and were then stored at −80°C to await further analysis in total RNA extraction.

### Gene cloning

Primer sequences for the amplification of the CXCL12 coding sequence were designed as follows: Forward-5' ATGGACGCCAAGGTCGTCG 3' and Reversed-5' TTACTTGTTTAAAGCTTTCTCCAGG 3'. Total RNA was extracted from tree shrew's brain tissues using the Total RNA Isolation Reagent (Pufei Biology Co., Ltd, China mainland). The first-strand cDNA was synthesized using Oligo (dT) 18 primer (PrimeScript® RT, TaKaRa, Japan). The polymerase chain reaction (PCR) program was proceeded as follows: 1 cycle of 94°C for 3 min, 40 cycles of 94°C for 1min, 57°C for 30 s, and then 72°C for 30 s, followed by one cycle of 72°C for 10 min. Agarose-purified PCR fragments were further cloned into pMD-19T vector (TaKaRa, D102A, Japan) and transfected into DH5α competent *Escherichia coli* (Transgen bioscience, China mainland). Correct clones were finally identified by plasmid-DNA sequencing. The studied tree shrew CXCL12 cDNA sequence was deposited in GenBank (accession number: KF640640).

### Real-time PCR

Real-time PCR primers were designed using Primer 5.0 as follows: CXCL12: Forward- 5' ACAGATGTCCATGCCGATTC 3'and Reversed-5' GTTCTTCAGCCTTGCCACAA 3'; CXCR4: Forward-5' AATCTTCCTGCCCACCATCT 3' and Reversed-5' GGTGCAGCCTGTACTTGTCC 3'; 18s RNA: Forward-5' CAGCCACCCGAGATTGAGCA 3', Reversed-5' TAGTAGCGACGGGCGGTGTG 3'. Real-time PCR was conducted following the standard protocols using SYBR® *Premix Ex* Taq (TaKaRa, Japan): 1 cycle of 95°C for 2 min, 40 cycles of 95°C for 30 s, and 58°C for 40 s, followed by melt curve analysis. The expression levels of tree shrew CXCL12 and CXCR4 genes were normalized by housekeeping gene 18s RNA.

### Enzyme-linked immunosorbent assay (ELISA)

Serum was separated after blood had been clotted for 2 hours at room temperature (RT). Two mouse monoclone antibodies, anti-mouse (M) and anti-rat (R) CXCL12 antibodies (Elisa Biotech Co., Ltd, China mainland), were used to detect the CXCL12 concentration in tree shrew serum. ELISA was conducted according to the standard procedures provided by the manufacturer. A micro-plate reader (SpectraMax®340PC, USA) was used to read the optical density at 450 nm.

### Flow cytometry

Peripheral blood lymphocytes were isolated using mouse Ficoll-Hypaque gradient centrifugation (Beijing Solarbio S&T Co., Ltd., China mainland). Mouse anti-human CD184-congregated with PE, Mouse IgG2a, κ Isotype-congregated with PE, Rat anti-mouse CD184-congregated with FITC, and Rat IgG2b κ Isotype-congregated with FITC were used for flow cytometry. All flow cytometry antibodies were purchased from BD Biosciences (USA). Briefly, 1×10^6^ lymphocytes per 100 µl were incubated with a monoclone antibody for 20 min at room temperature. Cells were washed twice with phosphate buffer solution (PBS) and resuspended in 0.2 ml final volume of PBS. Flow cytometry was analyzed using a FACSVantage (BD Biosciences, USA) and data were analyzed using FlowJo (Tree Star, USA).

### *In vitro* transmigration

Transmigrations were conducted with AP48 48-well Boyden Chambers (Neuro Probe, Inc. USA) according to the protocols that are provided by the manufacturer. The chemotaxis chambers had a 5 mm diameter polycarbonate film with 5 µm pore. We seeded 2×10^4^ of lymphocytes into the top wells. To induce the cell migration, a recombinant human CXCL12 protein (R&D Systems, USA) with a broad concentration range from 0 ng/ml to 1000 ng/ml was added into the lower chambers. CXCR4-specific antagonist. AMD3100 (Sigma, USA) was used to inhibit the induced CXCL12-binding transmigration. AMD3100 was dissolved in dimethylsulfoxide (DMSO) (Beijing Solarbio S&T Co., Ltd., China mainland) to prepare 1 mg/ml of stock concentration and was stored at 4°C. The inhibitory effect of AMD3100 was tested at the concentrations ranging from 0.1 µg/ml to 100 µg/ml.

### Mobilization of hematopoietic progenitor cells *in vivo*

AMD3100 was dissolved in PBS. Tree shrews received a single tail-vein injection of AMD3100 at the dosage of 5 mg per kg. Peripheral blood was withdrawn at time intervals (0, 0.5, 1 and 2 h) after AMD3100 administration. Cells were further isolated by using mouse Ficoll-Hypaque gradient centrifugation (Beijing Solarbio S&T Co., Ltd., China mainland). Mouse anti-human CD133 antibody- and Isotype-conjugated with PE dye (Miltenyi Biotec, German) were used for the FASC analysis. Briefly, 1×10^6^ cells per 100 µl were incubated with antibody for 30 min at room temperature. Cells were washed twice with PBS and resuspended in 0.2 ml final volume of PBS. FACS was conducted to determine the percentage of CD133+ cells in tree shrews' peripheral blood.

### MTT cell survival assay

Lymphocytes were seeded in 96-well plates at a density of 2×10^4^ cells per well (200 µl). Cells were cultured in complete M1640 medium (Hyclone, Brazil) at 37°C with 5 % CO2. AMD3100 was dissolved in DMSO. And the DMSO concentration in the DMSO group was kept consistent with the AMD3100 group. The effects of AMD3100 on the survival of lymphocytes, as determined by MTT were conducted at day 0, day 1, day 3, day 5, day 7, day 9, and day 11, respectively. Briefly, to each well was added with 40 µl of MTS solution (CellTiter 96 AQueous One Solution Reagent, Progma, USA) and cells were incubated for 4 h. Plates were read using a microplate reader (Bio-Rad, USA) at 490 nm absorbance.

### Bioinformatics

Amino acid sequences were aligned via ClustalX. Phylogenetic trees were constructed using the Neighbor-Joining Method and Maximum Likelihood in MEGA 5.0 with 1000 bootstrap replicates [52], [53]. GenBank accession numbers for all proteins are as follows: CXCL12 (human: AAH39893.1; monkey: NP_001028106.1; mouse: AAH06640.1; rat: AAH78737.1; horse: XP_005602693.1; pig: AAQ84094.1 squirrel: XP_005334466.1; chicken: NP_989841.1; frog: NP_001015764.1 and tree shrew: AHB11183.1) and CXCR4 (human: AAB81970.1; monkey: NP_001036110.1; tree shrew: AAO47588.2; mouse: NP_034041.2; Rat: NP_071541.2 horse: XP_005601526.1; pig: AAZ32767.1; squirrel: XP_005315892.1; chicken: NP_989948.2, and frog: NP_001090831.1). The CXCL12 and CXCR4 protein structures were generated via Homology Modeling using MODELLER [54]. Human CXCL12 and CXCR4 protein structures were exported as templates from the Protein Data Bank (PDB) (http://www.rcsb.org/pdb). The PDB ID of human CXCL12 was 2KEE [55] and human CXCR4 was 3OE8 [56]. The structural models of CXCL12 and CXCR4 in tree shrews were built using PyMol 1.5 [57].

### Statistics

Data were expressed as means±SEM (Standard Error of Mean). Statistical analysis was performed using two-tailed unpaired student's t test. *P*-values <0.05 were considered statistically significant those <0.01 were regarded as highly significant. All the experiments were performed in triplicate.

## Supporting Information

Figure S1 **Coding sequence of tree shrew's CXCL12 and its predicted amino acids.** Start code is underlined and stop code is marked by an asterisk.
(TIF)

Figure S2 **Coding sequence of tree shrew's CXCR4 and its predicted amino acids.** Start code is underlined and stop code is marked by an asterisk.
(TIF)

Figure S3 **AMD3100 did not significantly impair lymphocytes survival in tree shrews.** AMD3100 was dissolved in dimethylsulfoxide (DMSO) with the DMSO concentration in the DMSO group was kept consistent with the AMD3100 group. The relative cell number in each well was expressed as the absorbance values at 490 nm. The experiment was repeated in triplicate to ensure quality of the results.
(TIF)
