# Supplementary figures and images for: CXC Chemokine CXCL12 and Its Receptor CXCR4 in Tree Shrews (Tupaia belangeri): Structure, Expression and Function
Source: PLoS One. 2014 May 23;9(5):e98231. doi: 10.1371/journal.pone.0098231 (PMC4032326; doi:10.1371/journal.pone.0098231)

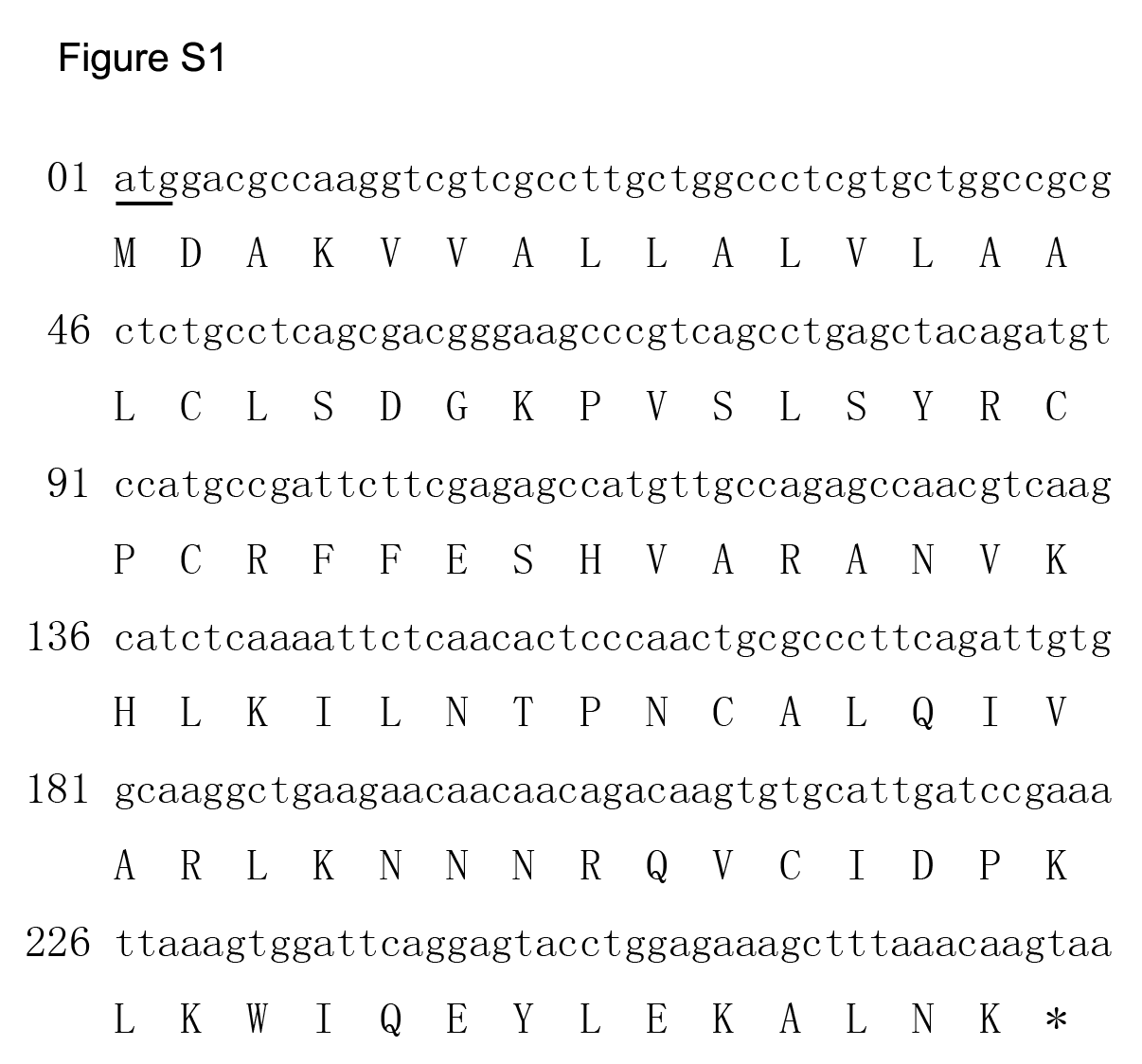

Supplement: Figure S1 — Coding sequence of tree shrew's CXCL12 and its predicted amino acids. Start code is underlined and stop code is marked by an asterisk. (TIF) [file pone.0098231.s001.tif]

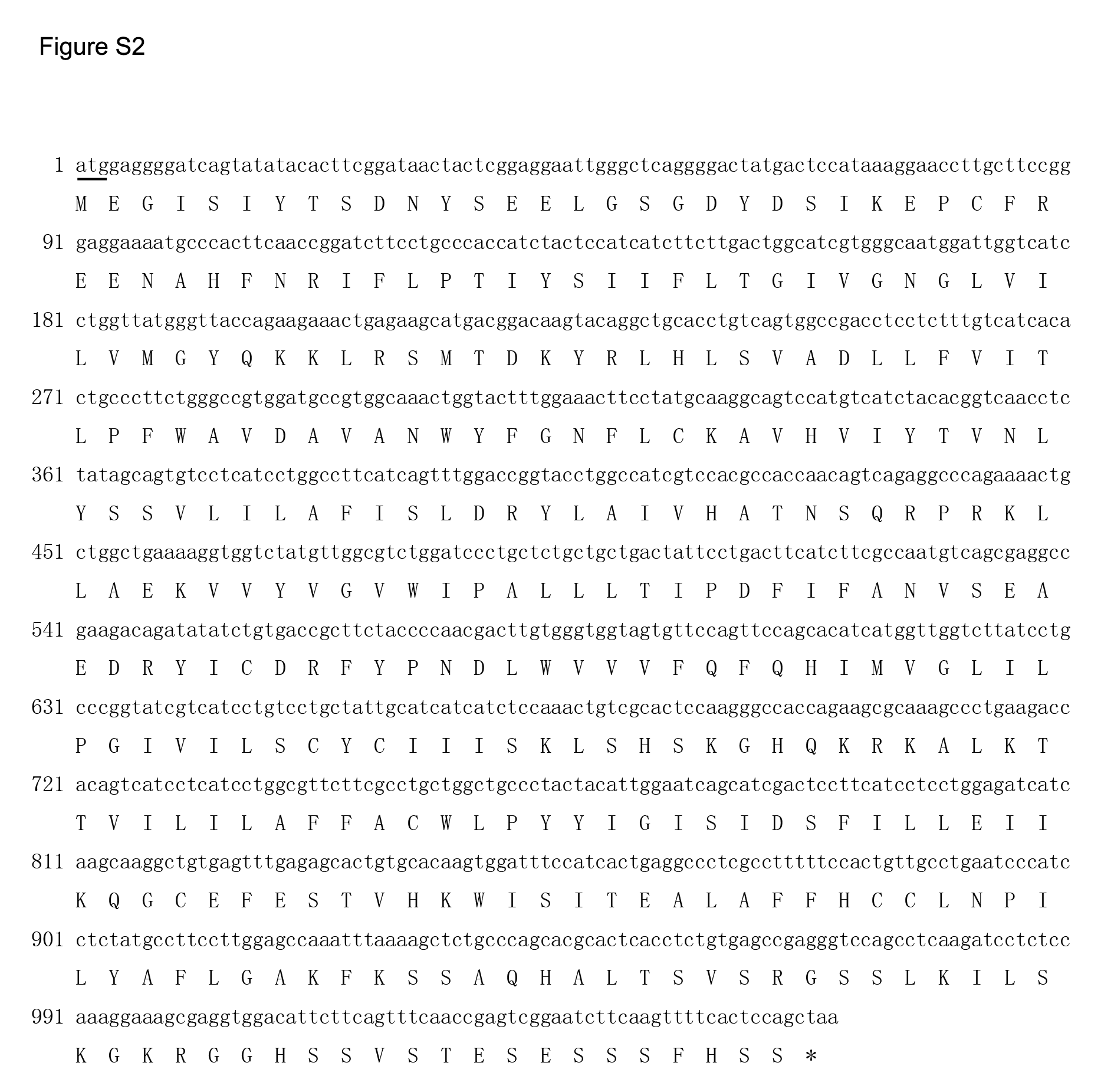

Supplement: Figure S2 — Coding sequence of tree shrew's CXCR4 and its predicted amino acids. Start code is underlined and stop code is marked by an asterisk. (TIF) [file pone.0098231.s002.tif]

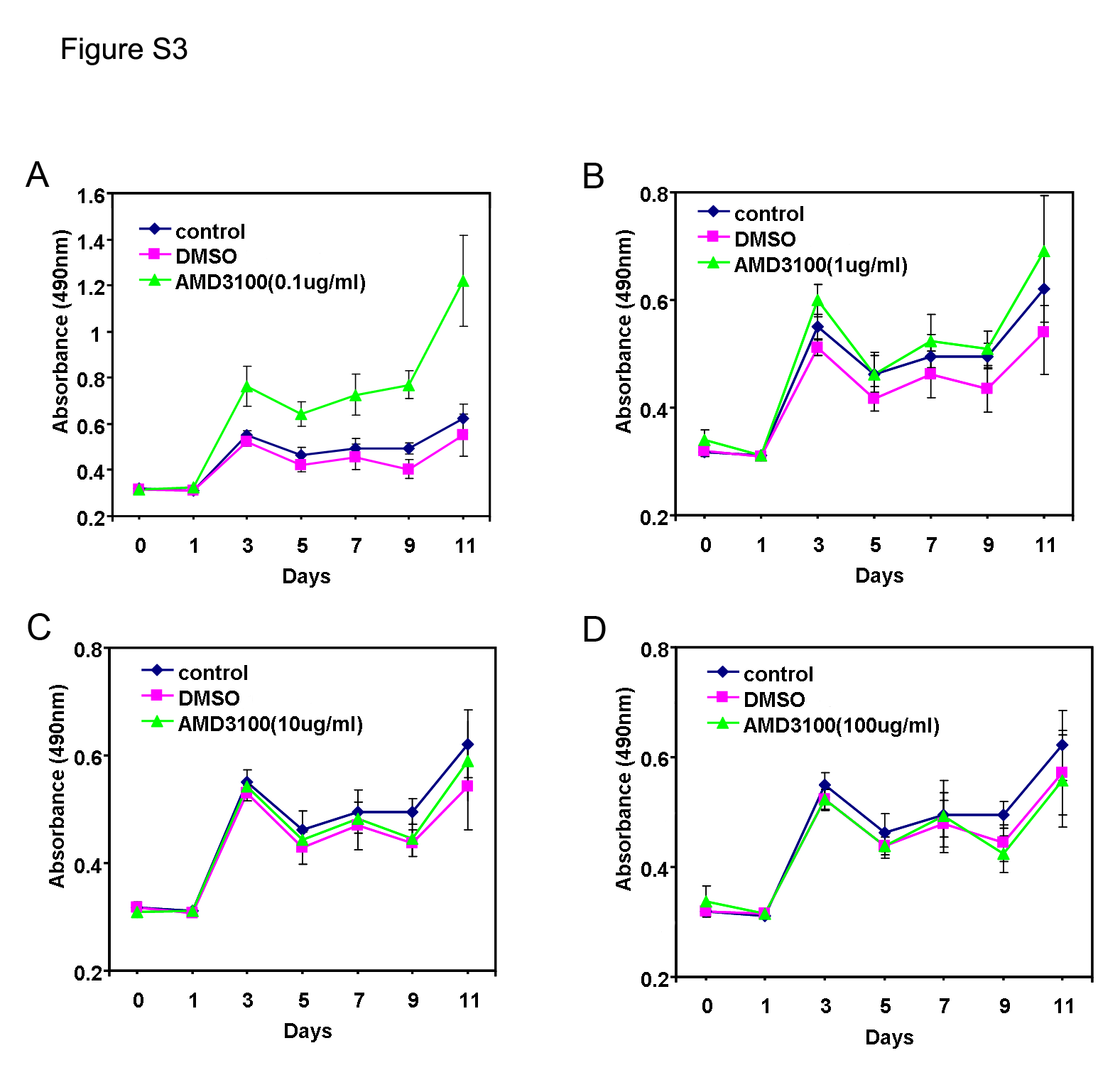

Supplement: Figure S3 — AMD3100 did not significantly impair lymphocytes survival in tree shrews. AMD3100 was dissolved in dimethylsulfoxide (DMSO) with the DMSO concentration in the DMSO group was kept consistent with the AMD3100 group. The relative cell number in each well was expressed as the absorbance values at 490 nm. The experiment was repeated in triplicate to ensure quality of the results. (TIF) [file pone.0098231.s003.tif]
